# MRI of endometrium cancer – how we do it

**DOI:** 10.1186/s40644-016-0069-1

**Published:** 2016-05-09

**Authors:** Matthias Meissnitzer, Rosemarie Forstner

**Affiliations:** Department of Radiology, Landeskliniken Salzburg, Paracelsus Medical University, Müllner Hauptstr. 48, 5020 Salzburg, Austria

**Keywords:** Endometrial cancer, Uterine neoplasm, Staging uterine cancer, Magnetic resonance imaging

## Abstract

Endometrial cancer is the most common malignancy of the female pelvis. New concepts in endometrial cancer treatment emphasize the value of MRI as a major predictor of lymph node metastasis and tumour recurrence. MRI findings aid in triaging patients for a more tailored therapeutic regimen.

This review discusses the value of MRI in the preoperative assessment of endometrial cancer and provides a practical approach how to image and report endometrial cancer. Practical tips are provided how to increase the diagnostic accuracy in staging of endometrial cancer and how to avoid pitfalls.

## Background

Endometrial carcinoma is the leading malignant tumour of the female genital tract in industrialized countries. Over the last decade the annual incidence has remained stable with an estimated 25.1 cases per 100. 000 women [1]. The vast majority of endometrial cancer is diagnosed at an early stage with atypical uterine bleeding in postmenopausal age. The 5-year overall survival is 81.7 %, but it varies broadly from 20 to 91 % for different tumour histologies and stages [2, 3]. For radiologists it is important to incorporate the histopathological subtypes I or II in their reporting. These subtypes differ not only with regard to histology and risk factors, but also in clinical features, including stage at presentation, risk of dissemination and in recurrence rate. Type I accounts for 80–85 % of endometrial cancers, it is estrogen-responsive and has a favorable prognosis [4]. Histologically it constitutes endometrioid adenocarcinomas grade I and II. Endometrial cancer type II is characterized by rapid tumour progression and a biological behavior often similar to ovarian cancer. Histologically it comprises endometroid cancer grade 3, and other rare histologies, e.g. serous cancers, clear cell cancers and carcinosarcoma/mixed Müllerian tumours [5]. Surgical staging with total abdominal hysterectomy and bilateral salpingo-oophorectomy has been the mainstay of therapy in endometrial cancer. Findings at staging also guide subsequent adjuvant treatment. There is ongoing controversy on the value of routine pelvic and para-aortic lymphadenectomy in early endometrial cancer surgery [5]. Recently a trend towards tailored lymphadenectomy is seen in many cancer centers, as it has been shown that only patients with intermediate or high risk endometrial cancer benefit from pelvic and paraaortic lymphadenectomy [5, 6].

## Review

### Role of MRI in the diagnostic work-up of endometrial cancer

Internationally, the practice of preoperative MRI evaluation of patients with endometrial cancer differs widely. According to the American College of Radiology (ACR) appropriateness criteria “MRI should be the preferred imaging modality for treatment planning, when available”, as it allows best overall assessment of the disease [7]. The National Comprehensive Cancer Network (NCCN) guidelines advise MRI when cervical invasion is suspected but also in pre-treatment evaluation of type II endometrial cancer [8]. The European Society of Urogenital Radiology guidelines recommend MRI in high and intermediate risk cancers, in suspected advanced disease and before lymph node sampling [9]. In 2015 a multidisciplinary European expert consensus meeting on endometrial cancer advised MR imaging in apparent stage I endometrial cancer to assess the depth of myometrial invasion, when tailored lymph node dissection is performed. However, alternatively, expert ultrasound (US) and/or intraoperative pathological exams are other options [5]. Patients can be divided into three risk categories based upon histopathological tumour type and grade and depth of myometrial invasion [10]. There is increasing evidence that when findings of staging MRI and hysteroscopic biopsy are combined, women at high risk of lymph node metastases can be identified preoperatively [10–13]. In one study this yielded an accuracy of 81 % and was superior to combined transvaginal sonography (TVS) and hysteroscopic biopsy [13]. Another central preoperative finding in MRI is cervical stromal invasion. This requires modification of the surgery technique including radical hysterectomy and pelvic and abdominal lymphadenectomy. Combined radiotherapy treatment is performed in most centers [5, 11]. MRI findings also contribute in triaging and guiding neoadjuvant therapy in advanced endometrial cancer in multidisciplinary consensus conferences [5, 11].

### Indications

At our institution MRI indications to assess endometrial cancer include: histologically proven endometrial cancer with histologies or US findings suggesting intermediate or high risk, sonographically suspected endometrial cancer and vaginal stenosis (without access for biopsy), uterine cancer of unknown origin (endometrial versus endocervical), central pelvic mass likely malignant, rapidly enlarging uterus in postmenopausal age, and in advanced metastatic cancer spread and suspected uterine neoplasm in CT.

### Imaging

#### Imaging technique

Measures undertaken to reduce bowel motion and thus improve image quality include fasting for at least 4 h before the exam, injection of antiperistaltic drugs (hyoscine butylbromide preferably intramuscularly or glucagon intramuscularly) before the exam, and tight wrapping of a belt around the pelvis and abdomen. Anxious patients are positioned with feet first. As a full bladder may cause artifacts and is barely tolerated by most patients, the bladder should be emptied at about 30 min or just before the exam.

**Fig. 1:**
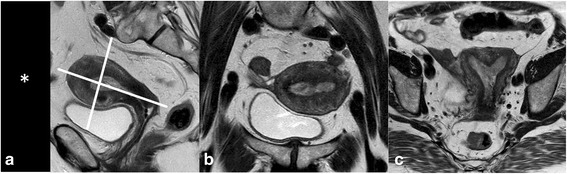
Basic imaging technique in stage Ia endometroid cancer. Anterior sat bands (*) are used to reduce artifacts from breathing. Sagittal T2WI (**a**) render an overview of the pelvis including the distal lumbar region. The image displaying the distended uterine cavity is used as reference for sections perpendicular (**b**) and along the long uterine axis (**c**). DWI are used in same plane as in b. The tumour displays intermediate SI on T2WI and shows restricted diffusion

A phase array coil is used for pelvic and abdominal imaging. Correct positioning of the coil with regard to pelvic anatomy is pivotal, especially when small FOV and fat saturation techniques are used. Fat saturation bands are applied to eliminate motion artifacts from the anterior abdominal wall (Fig. 1).

**Fig. 2:**
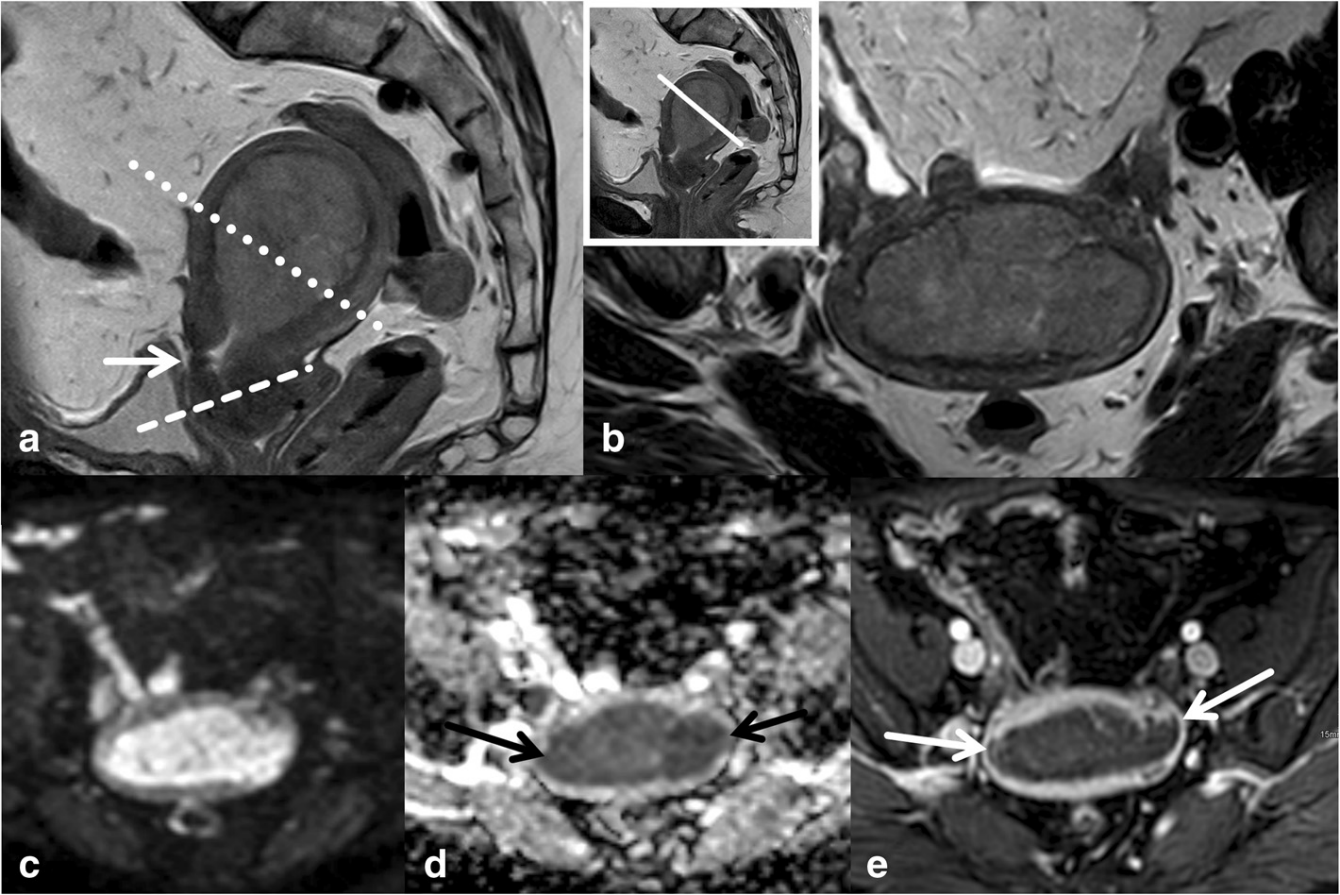
Value of complementary DWI and GdT1 to assess myometrial invasion. Endometrial cancer expanding the uterine cavity and extending to the upper cervix (arrow) is seen on sagittal T2WI (**a**). The planes for assessing myometrial and cervical invasion are perpendicular to the long axis (dotted and dashed lines, **a**). T2WI perpendicular to the uterine cavity (**b**) demonstrates thinning of the myometrium. Deep myometrial invasion can be confirmed by DWI (b = 1000) (**c**) and (**d**) and by Gd T1 FS (**e**)

A localizer in 3 planes provides anatomical details, particularly position and size of the uterus and serves for planning the diagnostic images. The key imaging sequence to assess the uterus is T2WI. Thus, 3 planes of T2WI will allow definition of size, location, extent, and morphology of endometrial cancer. This is best done by choosing a sagittal T2WI displaying the tumour within the uterine cavity or the dilated uterine cavity. Two additional planes parallel and perpendicular to the uterine cavity are then performed (Fig. 1). In case of tumour extending to the cervix a complementary section perpendicular to the cervical canal should be included. This serves to assess cervical stromal extension, and is necessary in small tumour size or equivocal findings. If the tumour is unequivocally thinning the cervix already in the sagittal view, such a plane can be omitted. Transaxial T1WI facilitate depiction of pelvic lymph nodes and assist in defining the quality of fluid filled distension of the uterine cavity and the morphology, particularly of hemorrhagic tumour components. Our routine protocol always includes DWI of the pelvis, as this technique is fast and will render valuable information for tumour detection, differentiation of tumour from benign lesions and will alert to pelvic lymph nodes as well as tumour spread beyond the uterus. The sequence should be acquired in the identical angulation as T2WI (Fig. 2). Some authors advise DWI in 2 planes, as these will allow both complete coverage of the tumour and the pelvis including the pelvic lymph node status [14]. Lymph nodes can easily be detected on DWI but the DWI/ADC can’t distinguish between benign and malignant lymph nodes, additional morphologic criteria are needed for this differentiation. The value of Gd T1WI is problem solving in equivocal findings of tumour spread, typically of the depth of myometrial invasion and of presence of cervical invasion. If the study is performed under radiologist supervision and if based on the basic sequences T2WI and DWI tumour extension can be unequivocally defined additional GdT1WI is not necessary. Otherwise, dynamic contrast enhancement sequences are performed to improve the conspicuity of tumour extension and presence of metastases. Gadolinium improves tumour –myometrium discrimination, with best contrast gained at about 120–180 s after intravenous (IV) contrast application (9) (Fig. 2). Optimal cervical contrast may be acquired in a later phase with a delay of 4 min after IV contrast media [15]. Of note, in advanced peritoneal disease and ascites Gd T1WI should not be performed with a delay longer than 5 min as ascites may show delayed contrast uptake and thus peritoneal metastases may be obscured [16]. Retroperitoneal nodes, kidneys and liver should also be included in the staging exam. We perform transaxial DWI and Gd FS T1WI if contrast media were administered, otherwise T2STIR sequences. Technical details (3 T) of the staging protocol are summarized in Table 1.

**Table 1:** MR protocol for evaluation of endometrial cancer

	Pelvic series							Abdominal series		
Sequence	Sagittal T2W	Oblique transaxial T2W	Oblique coronal T2W	Trans-axial T1W	Trans-axial DWI	Gd DCE 3D Ultrafast spoiled GE	Gd Dixon 3D FSPGR	T2 FS	DWI	Gd Dixon 3D FSPGR
Range	pelvis	pelvis	pelvis	pelvis	pelvis	pelvis	pelvis	abdomen	abdomen	abdomen
Sections	26	29	29	27	28	55/slab	150	46	38	150
TR	3100	3500	3500	540	3195	3	3,7	2177	4429	3,2
TE	100	90	90	10	63	1,42	1.36/2.4	75	59	1.15/2.1
Angulation/recon-struction		perp.^a^	parallel ^a^		perp^a^	sagittal	transaxial			transaxial
Slice thickness (mm)	3,5	3	3	6	5	4	1.5	5	8	1,5
Gap (mm)	0,35	0,3	0,3	6	0,5			0,5	0,8	
FOV (mm)	260	210	210	250	300	250	350	280	300	350
B values					0,800,1200				0,800,1200	
phases						4: un-enhanced, at 1, 1.45 and 2.10 min				
Matrix	580 × 260	280 × 261	280 × 261	276 × 257	100 × 98	168 × 167	236 × 158	252 × 168	116 × 124	236 × 158
Acquisition time (min)	3:36	3:30	3:30	2:16	3:28	15 s each	22 s	3:30	3:40	0:19

^a^Angulation along uterine axis

NB: In pelvic and abdominal series contiguous coverage is necessary

### Imaging findings

#### Staging of endometrial cancer

Endometrial cancer is staged surgico-pathologically according to the FIGO system, the world wide most commonly used classification, or the TNM system. The 2009 revised FIGO staging classification has facilitated radiological analysis of tumour spread within the uterus as major challenges for radiological assessment have been eliminated [17, 18]. The recent FIGO system and corresponding MRI findings are summarized in Table 2.

**Table 2:** FIGO Staging of endometrial carcinoma and adapted MRI findings^a^

FIGO Stage	Tumour invasion	MR imaging findings
I	Tumour confined to uterus	
IA	≤50 % of myometrium	Abnormal SI in endometrial cavity or confined to inner half of myometrium
IB	>50 % of myometrium	Extends into the outer half of myometrium
II	Cervical stromal invasion, but not extension beyond uterus	Disruption or focal thinning of cervical stroma
III	Local or regional spread	
IIIA	Serosa of uterus and/or adnexa	Disruption or irregular uterine contour caused by tumour; ovarian nodular tumour
IIIB	Vagina or parametria	Direct tumour extension of upper vagina or/and parametrial tissues
IIIC	Metastases to pelvic and/or paraaortic lymph nodes	
IIIC1	Pelvic nodes	Lymph nodes >8 mm in short axis
IIIC2	Paraaortic nodes w/wo pelvic nodes	Lymph nodes >10 mm in short axis
IV	Bladder/bowel mucosa; or distant metastases	
IVA	Bladder/bowel mucosa	Tumour disrupts bladder or bowel muscle and invades mucosa; not bullous edema
IVB	Metastases abdominal and extraabdominal; inguinal lymph nodes	Tumour deposits at distal sites including peritoneal mets, bladder, bone liver mets, and distal lymph node metastases

^a^Adapted from FIGO Commitee on Gynecologic cancers. FIGO classification of cancer of the vulva, cervix, and corpus uteri. International Journal of Gynecology Obstetrics 2014; 115:97–98

**Fig. 3:**
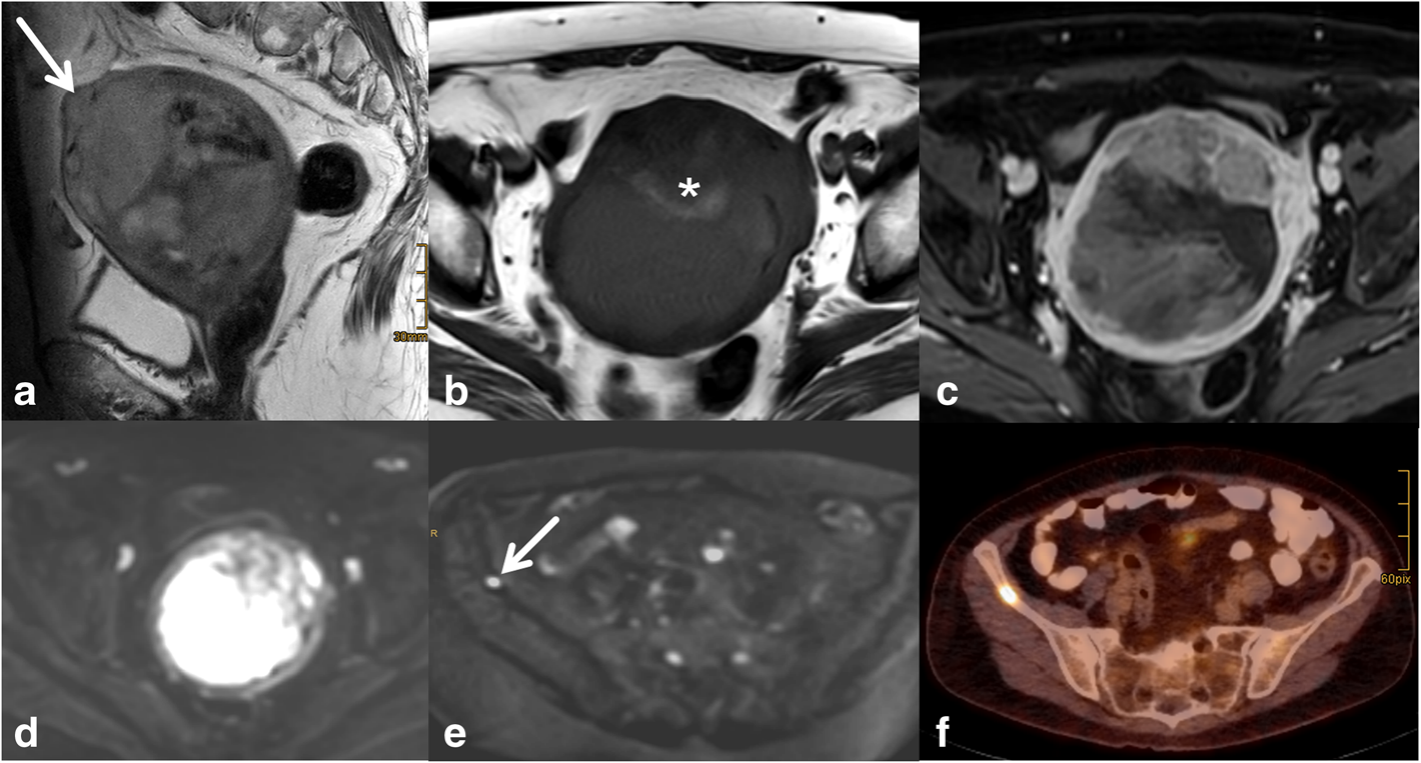
Subserous tumour spread. Type II endometrial cancer (grade III) with subserous uterine growth (**a**, arrow). Transaxial T1WI demonstrates hemogeneous structure with hemorrhage (**b**,*). Necrosis is seen on T1GD FS (**c**). Transaxial DWI (**d**) at the level of the primary tumour and above (**e**) demonstrate high signal on b 1000. The focal osseous lesion with high signal on high b value image corresponds to a bone metastasis in PET/CT (**f**)

**Fig. 4:**
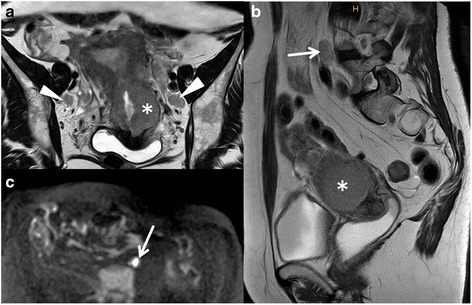
DWI to depict lymph nodes. Cancer of the endometrium originating from the distal corpus uteri with growth to the cervix (**a** and **b**,*). Cervical stromal thinning displayed in **a**. Bilateral regional lymph nodes are well displayed on T2WI **a**, arrowheads) and on DWI. The lymph node at the level of the aortic bifurcation (arrow) on sagittal T2WI (**a**) displaying high SI on DWI (**c**) was not metastatic at histology

Depth of myometrial invasion (less or ≥ than 50 % myometrial invasion) divides stages IA from stage IB (Figs. 1 and 2). Tumour growth along the endocervix but without cervical stromal extension is also defined as stage I. For assigning stage II cervical stromal invasion must be present. Stage IIIA defines tumour spread to adjacent uterine serosa or adnexa (Fig. 3). In Stage IIIB vaginal or parametrial involvement is present. Stage IIIC is characterized by lymph node metastases, either in the pelvis (IIIC1) or in the paraaortic region (IIIC2) (Fig. 4). Tumour invasion into bladder or bowel mucosa classifies as stage IVA. Stage IVB is classified in distant metastases or inguinal lymph node involvement.

#### Key points in MR staging of endometrial cancer

**Fig. 5:**
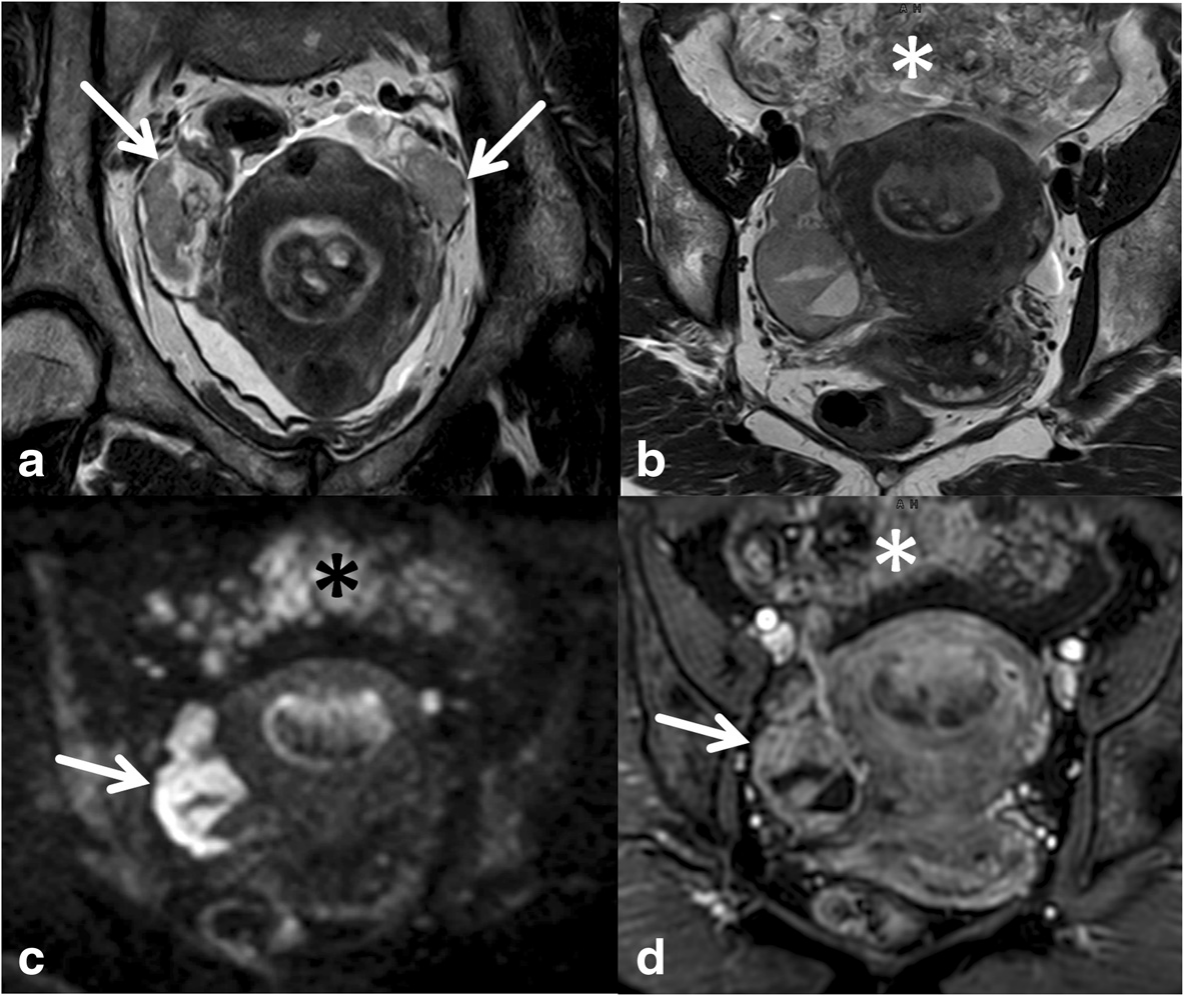
Peritoneal spread in aggressive tumour histology. A broad-based endometrial mass is seen on transaxial and coronal T2WI (**a**, **b**). Gd T1WI also demonstrates bilateral solid enhancing adnexal lesions (arrows, **d**) with only moderate ovarian enlargement. Further, omental nodules are displayed (*). On the high b value image DWI demonstrates high signal intensity of the ovarian mass (arrow, **c**) and the peritoneal deposits (*, **c**). The histology of type II endometrial cancer favours metastases. However histopathological proof is necessary for differentiation

- Endometrial cancer is typically mildly hyperintense on T2-WI compared to normal myometrium [19] (Fig. 1). Subtype II tends to exhibit inhomogeneous morphology with areas of haemorrhage and necrosis and is commonly diagnosed with deep myometrial invasion (Fig. 3).
- Most tumours arise from the fundus and display exophytic expansion. Diffuse infiltrative growth is rarely found and characterized by diffuse myometrial thickening.
- Evaluation of the uterus at least in 2 planes using T2WI (maximal 4 mm slice thickness) along the uterine axis is mandatory to define the depth of myometrial invasion [9].
- Multiparametric approach combining T2WI, DWI and dynamic Gd MRI will render the most comprehensive approach to assess local tumour spread. It is most useful if radiologist supervision when images are obtained is not feasible or in less experienced readers (Fig. 2).
- Cervical invasion is assumed when there is thinning or focal disruption of the hypointense signal of the cervical stroma and its continuity with the tumour. Stromal invasion, particularly when subtle is best identified in a plane perpendicular to the cervical canal.
- Invasion of the pelvic wall is suggested when the distance between the tumour and the pelvic wall including internal obturator muscle, levator ani, piriformis muscle or iliac vessels is less than 3 mm.
- Rectum or bladder wall invasion is best evaluated in the sagittal plane. Preservation of the fat plane between tumour and bladder or rectum allows reliable exclusion of stage IVA.
- In aggressive tumour histologies (type II) careful assessment of the abdomen and pelvis for peritoneal deposits is warranted (Fig. 5).

#### Key points in lymph node imaging in endometrial cancer

- Regional nodes are pelvic and paraaortic lymph nodes. The latter may be the first manifestation of lymphatic spread.
- The rate of lymph node metastases in low risk cancers is very low (2.4 %). It rises with increasing risk categories to 9 % resp. 24 % in intermediate resp. high risk categories [5 ,11].
- Lymph nodes are easiest to depict on DWI due to their high SI on the high b value image.
- As DWI is limited in predicting lymph node metastases classical morphological criteria have to be combined for characterization (Fig. 4). These include short axis diameter of pelvic lymph nodes with > 8 mm and abdominal lymph nodes > 10 mm. However, smaller lymph nodes with low ADC, irregular contours, with necrosis and clusters of lymph nodes may also be called suspicious of metastases [20].

#### Pitfalls, challenges and how to overcome these

- Tumour involving more than half of the myometrial thickness is staged as IB. The area of the uterine cornua is physiologically thinner than the normal myometrium. Hence, particularly, when it is symmetrical invasion at this location should not be overcalled.
- In atrophic uteri and in tumours isointense relative to myometrium complementary problem solving modalities should be performed complementary to T2WI. In DWI the angulation along the uterus should be identical to the T2W sequence. High resolution 3D T1 WI at about 2–2.5 min after IV contrast media application may be best able to solve the problem [9].
- If the depth of myometrial invasion is equivocal on T2WI and DWI, complementary GDT1 WI should be applied [15] (Fig. 2).
- In thinning of the endometrium caused by tumouros distension of the uterine cavity by expansively growing cancers myometrial assessment may be challenging [21]. Symmetry and smooth contours favour stage IA.
- In endometrial cancer and coexisting adenomyosis or atypical uterine leiomyomas the complementary use of both DWI and Gd is advised to assess the depth of myometrial invasion. Of note, both adenomyosis and some leiomyomas may show restricted diffusion, and leiomyomas may also be hypervascular.
- Double angulation technique provides a true orthogonal view of the uterus and may improve assessment of myometrial invasion in rotated or tilted uteri. Thus, the problem of volume averaging artifacts can be reduced [15].
- In equivocal lymph nodes morphologic criteria should be combined with functional information. Enhancement pattern or ADC similar to the uterine cancer support the diagnosis of metastasis
- To reduce pitfalls in interpretation in DWI the findings should always be correlated with T2WI. The optimal high b value varies from field strength and vendors. It should be 800 mm/s [2] or more and is optimal when the fluid in the urinary bladder appears dark.

#### Clinically challenging constellations and how to aid with imaging

##### Synchronous endometrial cancer and adnexal masses

Estrogen stimulation of the endometrium is a major risk factor for type I endometrial cancer. Thus hormone- active ovarian tumours and endometrial hyperplasia or endometrial cancer may be detected simultaneously. Coexisting endometrial cancer is reported in granulosa cell tumours and thecomas in 3–25 % of cases [22]. In imaging granulosa cell tumours may display a wide range from solid heterogeneous to multicystic masses. In contrast, when thecomas display typical imaging features of well-delineated solid masses with low SI in T2WI MRI allows a specific diagnosis [23].

More challenging is the constellation of endometrial cancer and imaging features of a malignant ovarian mass. This may present both an independent ovarian neoplasm or metastases from endometrial cancer. Synchronous endometrial and epithelial ovarian cancer are reported in 5 % of patients with endometrial cancer and in 10 % of patients with ovarian cancer [4]. In endometroid cancers, synchronous ovarian cancers are more likely to occur in premenopausal age and often these may only be microscopic. The rare clear cell ovarian cancer typically presenting as a cystic mass with solid nodules protruding into the lesion may also be associated with endometrial cancer [24]. In general, metastases to the ovaries seem more likely to be found in type II endometrial cancer [25] (Fig. 5). Further, metastases from endometrial cancer rather than a second primary in the ovaries should be suspected in bilateral ovarian involvement in small size of the ovarian mass or in multinodularity of the ovaries [26].

##### Uterine cancer of unknown origin- endometrial or cervical cancer

**Fig. 6:**
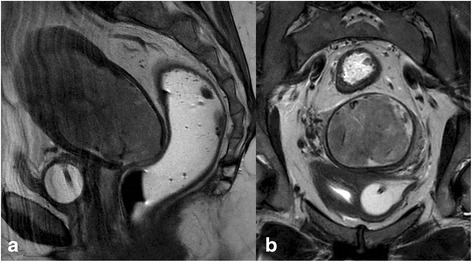
Clinically adenocancer of unknown origin. Sagittal T2WI (**a**) shows a large tumour with the epicenter in the uterine corpus extending to the external cervical os. Circumferential thinning of the cervical stroma is seen on the oblique transaxial section (**b**). There is no evidence of involvement of the uterovesical ligaments or the bladder wall. All these findings favour the diagnosis of endometrial rather than cervical cancer

Usually prior to MRI staging the origin of a uterine malignancy is known based upon clinical assessment and/or obtained histology. However, in a small subset of patients, e.g. one institution reported 3.2 %, this remains uncertain [27]. At histology and even immunohistochemically the differentiation of endometrial from endocervical carcinoma may sometimes be challenging [28]. In this constellation Radiology can assist in defining the origin by analyzing imaging features of the tumour and its local patterns of growth. Clinically, this information is pivotal as treatment regimen differ completely. In one study differentiation was feasible in 85 % (45/48) of cases [29]. Features favouring endometrial cancer over cancer of the uterine cervix are: epicenter of the mass is the endometrial cavity rather than cervix, depiction of tumour growing within the endometrial cavity or hypovascularity in arterial phase in small tumour size [28] (Fig. 6). However, small cancers, type II cancers or sarcoma may be hypervascular, but the latter usually tend to be heterogeneous. Central necrosis and per continuation bladder invasion or uterovesical fistulation favour the diagnosis of cervical cancer.

#### Image interpretation

##### Reporting checklist

Radiology reports may be provided in a structured computer assisted report or on a free text basis. Regardless of the format the following central elements have to be addressed: Lesion size, using diameters at least assessed in two planes, size of the uterus in two orthogonal planes and distension of the uterine cavity; characteristics of the tumour (e.g. heterogeneity, haemorrhage, necrosis, hypervascularization). Further, extension of the tumour within the uterus (less or more than half of the myometrium and cervical stroma) and extension to uterine surface or ovaries have to be notified. Bladder and particularly rectosigmoid colon invasion should be addressed. The status of lymph nodes should be assessed. This includes allocation and size of suspicious lymph nodes in the pelvis and retroperitoneum. Other tumour-related findings to mention include distant metastases (e.g. peritoneal spread liver metastases and lymph nodes) and urinary obstruction. Finally, findings relevant for surgery, e.g. vascular variations of pelvic and retroperitoneal vessels and other findings including incidentalomas should be reported. Although a matter of debate among radiologists, at our institution we add the MRI stage as indicated by the findings in the end of the report [9].

## Conclusions

MRI is a potent imaging tool assisting to triage treatment in women with endometrial cancer based on multidisciplinary team consensus. The depth of myometrial invasion, cervical extension and lymph node metastases present major findings to analyse in the staging MRI. Thus, combined with the histological subtype MRI renders crucial information for pretreatment risk stratification. MRI assists in accurate treatment planning and selection of patients who will profit from paraaortic lymph node dissection. Radiologists have to be aware of the differences in imaging and clinical features of the two main types or endometrial cancer. Imaging technique can be optimized for endometrial cancer staging, and advanced techniques allow improved accuracy of local tumour spread. These may also alert to metastatic sites difficult to assess with conventional MRI.

## Abbreviations

ACR: American College of Radiology
ADC: Apparent diffusion coefficient
CT: Computed tomography
DCE: Dynamic contrast enhanced imaging
DWI: Diffusion weighted imaging
FIGO: International Federation of Gynecology and Obstetrics
Gd: Gadolinium
GE: Gradient echo
IV: Intravenous
MRI: Magnetic resonance imaging
NCCN: National Comprehensive Cancer Network
STIR: Short tau inversion recovery
PET/CT: Positron emission tomography–computed tomography
SI: Signal intensity
T: Tesla
TNM: TNM Classification of Malignant Tumours
TVS: Transvaginal sonography
US: Ultrasound
WI: Weighted imaging
